# Supplementary material for: Multifaceted Assessment of Porous Silica Nanocomposites: Unraveling Physical, Structural, and Biological Transformations Induced by Microwave Field Modification
Source: Nanomaterials (Basel). 2024 Feb 8;14(4):337. doi: 10.3390/nano14040337 (PMC10893391; doi:10.3390/nano14040337)
Supplement: Supplementary file 1 [file nanomaterials-14-00337-s001.zip › nanomaterials-2824914-supplementary.DOC.pdf]

nanomaterials

Supplementary data

Multifaceted Assessment of Porous Silica Nanocomposites: Unraveling Physical, Structural, and Biological Transformations Induced by Microwave Field Modification

Aleksandra Strach[1], Mateusz Dulski[2], Daniel Wasilkowski[3], Krzysztof Matus[4], Karolina Dudek[5], Jacek Podwórny[5], Patrycja Rawicka[6], Vladlens Grebnevs[7,8], Natalia Waloszczyk[8], Anna Nowak[3], P. Poloczek[2] and Sylwia Golba[2]

1. Doctoral School, University of Silesia, Bankowa 14, 40-032 Katowice, Poland; aleksandra.strach@us.edu.pl
2. Institute of Materials Engineering, University of Silesia, 75 Pulku Piechoty 1A, 41-500 Chorzow, Poland; mateusz.dulski@us.edu.pl; poloczekpaulina@gmail.com; sylwia.golba@us.edu.pl
3. Institute of Biology, Biotechnology, and Environmental Protection, Faculty of Natural Sciences, University of Silesia, Jagiellonska 28, 40-032 Katowice, Poland; daniel.wasilkowski@us.edu.pl; anna.m.nowak@us.edu.pl
4. Materials Research Laboratory, Silesian University of Technology, Konarskiego 18A, 44-100 Gliwice, Poland; krzysztof.matus@polsl.pl
5. Łukasiewicz Research Network, Institute of Ceramics and Building Materials, Cementowa 8, 31-938 Cracow, Poland; karolina.dudek@icimb.lukasiewicz.gov.pl; jacek.podworny@icimb.lukasiewicz.gov.pl
6. A. Chełkowski Institute of Physics, University of Silesia, 75 Pulku Piechoty 1, 41-500 Chorzow, Poland; patrycja.rawicka@us.edu.pl
7. Faculty of Chemistry, University of Latvia, Jelgavas Street 1, LV-1004 Riga, Latvia, vladlens.grebnevs@polsl.pl
8. Faculty of Chemistry, Silesian University of Technology, B. Krzywoustego Street 6, 44-100 Gliwice, Poland; natalia.szulc@polsl.pl

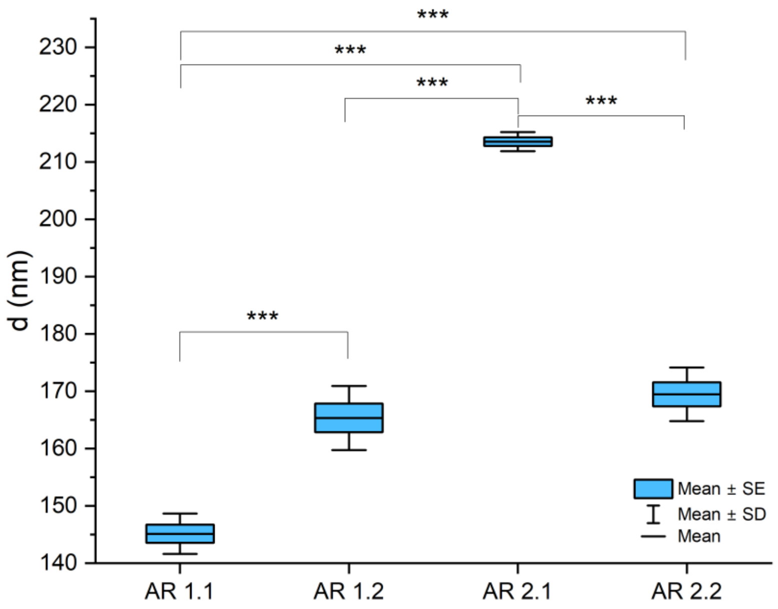

Fig. S1. Hydrodynamic averaged diameter (d_H) of silver-silica nanocomposites with individually microwave-treated silicas with statistically significant differences (*p < 0.05; **p < 0.01; ***p< 0.001). Data showed directly after sonication (t = 0 day).

Table S1. Values of diameter, polydispersity coefficient, and standard deviation were obtained for the tested samples at different sonification times.

Sonication time (min)	**AR 1.1**		**AR 1.2**		**AR 2.1**		**AR 2.2**	
	d ± Δd (nm)	PDI ± ΔPDI (-)	d ± Δd (nm)	PDI ± ΔPDI (-)	d ± Δd (nm)	PDI ± ΔPDI (-)	d ± Δd (nm)	PDI ± ΔPDI (-)
5	631 ± 14	0.68 ± 0.10	555 ± 23	0.65 ± 0.05	570 ± 30	0.50 ± 0.07	507 ± 16	0.45 ± 0.06
10	519 ± 6	0.56 ± 0.05	458 ± 14	0.44 ± 0.03	481 ± 20	0.43 ± 0.03	475 ± 15	0.42 ± 0.01
15	430 ± 5	0.42 ± 0.01	401 ± 6	0.40 ± 0.01	460 ± 20	0.43 ± 0.02	467 ± 15	0.49 ± 0.07
20	426 ± 14	0.50 ± 0.08	376 ± 14	0.39 ± 0.03	444 ± 6	0.47 ± 0.07	387 ± 10	0.39 ± 0.02
25	360 ± 9	0.40 ± 0.03	365 ± 7	0.38 ± 0.01	358 ± 13	0.40 ± 0.03	392 ± 7	0.43 ± 0.05

Table S2. Physicochemical properties of Ag-SiO_2 nanocomposites. d_H - hydrodynamic diameter, PDI - polydispersity index, and z - Zeta potential. The data are summarized with the standard deviation.

	d_H (nm)	PDI	z (mV)
AR 1.1	145.14 ± 3.53	0.40 ± 0.02	-42.06 ± 0.35

AR 1.2	165.34 ± 5.59	0.42 ± 0.01	-47.76 ± 0.67
AR 2.1	213.56 ± 1.65	0.38 ± 0.01	-47.22 ± 0.26
AR 2.2	169.46 ± 4.69	0.43 ± 0.01	-48.8 ± 0.84

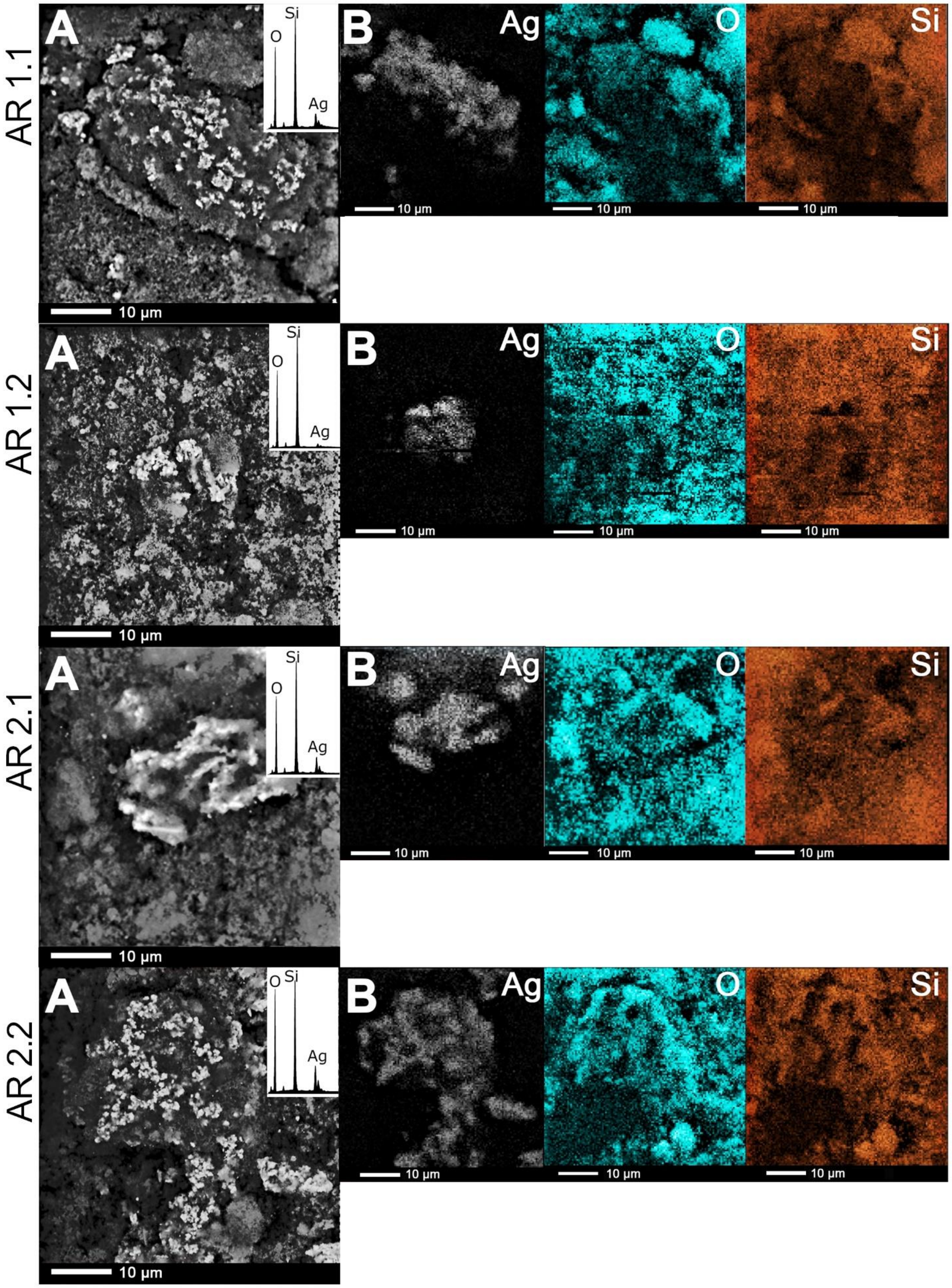

Fig. S2. SEM images with elemental distribution maps and SEM-EDS spectrum for silver-silica nanocomposites.

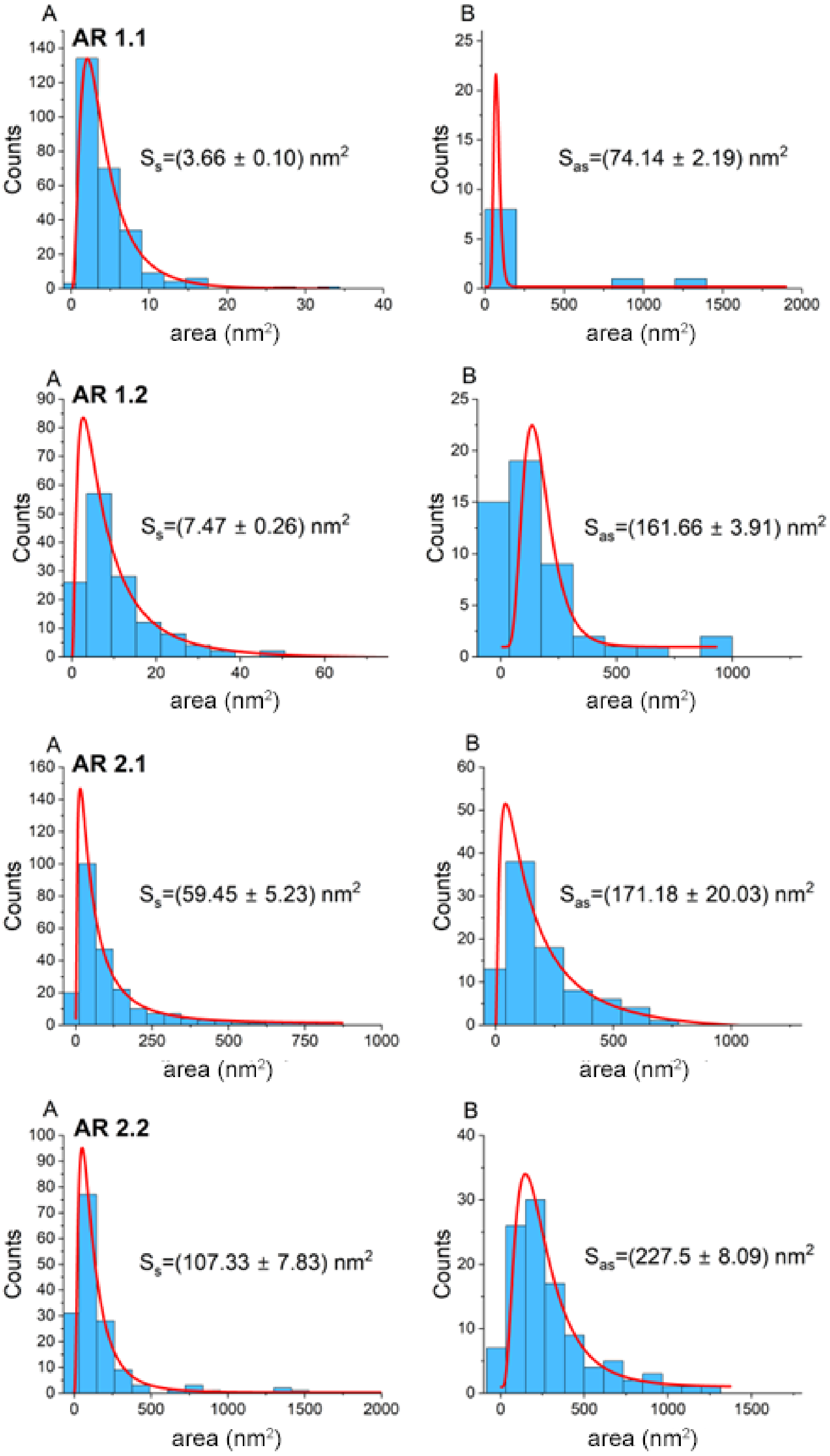

Fig. S3. The distribution of the area of spherical (S_s) and aspherical (S_{as}) Ag nanoparticles. The data on histograms were fitted with a logN function.
